# Untargeted Metabolomics Analysis Reveals Toxicity Based on the Sex and Sexual Maturity of Single Low-Dose DEHP Exposure

**DOI:** 10.3390/toxics11090794

**Published:** 2023-09-20

**Authors:** Hyeon-Jeong Lee, Jonghwa Jin, Yoondam Seo, Inseon Kang, Junghyun Son, Eugene C. Yi, Hophil Min

**Affiliations:** 1 Doping Control Center, Korea Institute of Science and Technology, Seongbuk-gu, Seoul 02792, Republic of Korea; hjl@kist.re.kr (H.-J.L.); mdsir@kist.re.kr (Y.S.); inseon61@kist.re.kr (I.K.); son.junghyun@kist.re.kr (J.S.); 2 Department of Molecular Medicine and Biopharmaceutical Sciences, Graduate School of Convergence Science and Technology, Seoul National University, Jongno-gu, Seoul 03080, Republic of Korea; 3 New Drug Development Center, Heungdeok-gu, Cheongju-si, Chungbuk 28160, Republic of Korea; jichang011@kbiohealth.kr

**Keywords:** DEHP, metabolomics, multivariate statistical analysis, LC–MS/MS, low dose

## Abstract

Di-(2-Ethylhexyl) phthalate (DEHP) is a prevalent environmental endocrine disruptor that affects homeostasis, reproduction, and developmental processes. The effects of DEHP have been shown to differ based on sex and sexual maturity. This study examines the metabolic profiles of mature adult rats from both sexes, aged 10 weeks, and adolescent female rats, aged 6 weeks, following a single 5 mg/kg of body weight DEHP oral administration. An untargeted metabolomic analysis was conducted on urine samples collected at multiple times to discern potential sex- and maturity-specific DEHP toxicities. Various multivariate statistical analyses were employed to identify the relevant metabolites. The findings revealed disruptions to the steroid hormone and primary bile acid biosynthesis. Notably, DEHP exposure increased hyocholic, muricholic, and ketodeoxycholic acids in male rats. Moreover, DEHP exposure was linked to heart, liver, and kidney damage, as indicated by increased plasma GOT1 levels when compared to the levels before DEHP exposure. This study provides detailed insights into the unique mechanisms triggered by DEHP exposure concerning sex and sexual maturity, emphasizing significant distinctions in lipid metabolic profiles across the different groups. This study results deepens our understanding of the health risks linked to DEHP, informing future risk assessments and policy decisions.

## 1. Introduction

Di(2-ethylhexyl) phthalate (DEHP) is commonly used to enhance the flexibility of polyvinyl chloride (PVC) products, such as food packaging, medical devices, and household items [1,2]. Because DEHP constitutes up to 40% of PVC plastic by weight and is not covalently bonded to plastic polymers, it can readily leach into the environment, becoming pervasive [1]. Consequently, DEHP is easily absorbed into the body through skin contact, ingestion, or inhalation, leading to various adverse effects, such as reproductive issues, hindering fetal growth [3], endocrine imbalances, miscarriages [4], and sexual differentiation disorders [5]. The current epidemiological study estimates that the total average daily dose of DEHP ranges from 17.03 to 24.54 µg/kg/day for children and adolescents in Eastern China [6].

Toxicological research on rats has indicated that DEHP inhibits steroidogenesis, resulting in anti-androgenic activity and reproductive toxicity in both sexes [7,8]. However, susceptibility to DEHP varies by age and sex. For instance, in a two-year repeated dose toxicity study, the limited observed adverse effect level (NOAEL) for kidney toxicity was 28.9 mg/kg/d for males and 36.1 mg/kg/d for females [9]. For testicular and developmental toxicity, NOAEL was 5 mg/kg/d in a three-generation reproductive toxicity study involving Sprague Dawley (SD) rats [10].

Exposure to DEHP during the peripubertal stage can cause sex-based differences in the liver and metabolic systems. There is delayed reproductive development in male rats and impaired thyroid metabolism in female rats [11]. Additionally, age plays a role in the testicular effects of DEHP. Previous studies have reported testicular damage in immature rats (25 days old) but not in mature ones (40 and 60 days old), indicating age-dependent toxicological responses [12]. Administering 10 mg/kg DEHP over four weeks led to early pubertal onset, increased testosterone serum, and seminal vesicle weights in three-week-old rats [13].

DEHP exposure during prenatal development can interfere with sex determination and lead to ovarian dysgenesis. These types of disruptions can profoundly affect female reproductive health and fertility [14]. Developmental exposure to DEHP has been found to compromise the endocrine functionality of the pancreas, potentially heightening the risk of diabetes or metabolic disorders later in life [15]. Exposure to DEHP can hinder follicle growth and decrease estradiol levels, impacting ovarian function and overall reproductive health [16].

Although the reproductive toxicity of DEHP is well-documented, its association with sex-specific neurotoxic effects is also noteworthy. Research has indicated that DEHP might differentially affect males and females in terms of their social communication capabilities and neural development [17]. For instance, male exposure to DEHP is linked with a heightened risk of autism, whereas females might manifest anxiety-like symptoms. These sex-specific neurotoxic effects underscore the importance of recognizing sex as a pivotal determinant when assessing DEHP’s neurological implications.

Recent advancements in metabolomics have been employed to decipher DEHP-induced metabolic disorders [18,19,20]. Specifically, studies utilizing liquid chromatography–mass spectrometry (LC–MS)-based metabolomics have highlighted the impact of dietary DEHP on energy-related metabolism, liver functionality [2], fatty acid metabolism [19], and DNA damage in rats [20]. However, most existing metabolomic investigations have focused on long-term exposures exceeding DEHP’s NOAEL levels [8,21]. To the best of our knowledge, an exhaustive metabolomic examination for single, low-dose, 5 mg/kg body weight DEHP exposure, which is the regulatory EU NOAEL of DEHP for developmental toxicity, centering on the acute effects tied to sexual maturity and sex, remains largely unexplored [22].

Based on a prior study, rats reach sexual maturity at approximately 7 weeks post-birth [23]. This research aims to assess the urine metabolite profiles of pre-mature adolescent female (6 weeks old), mature female, and male rats (10 weeks old) following exposure to low DEHP concentrations. Utilizing a mass spectrometry-based metabolomic technique, our goal was to identify and characterize metabolites, offering insights into the metabolic pathways impacted by DEHP exposure.

## 2. Materials and Methods

### 2.1. Study Design

**Figure 1:**
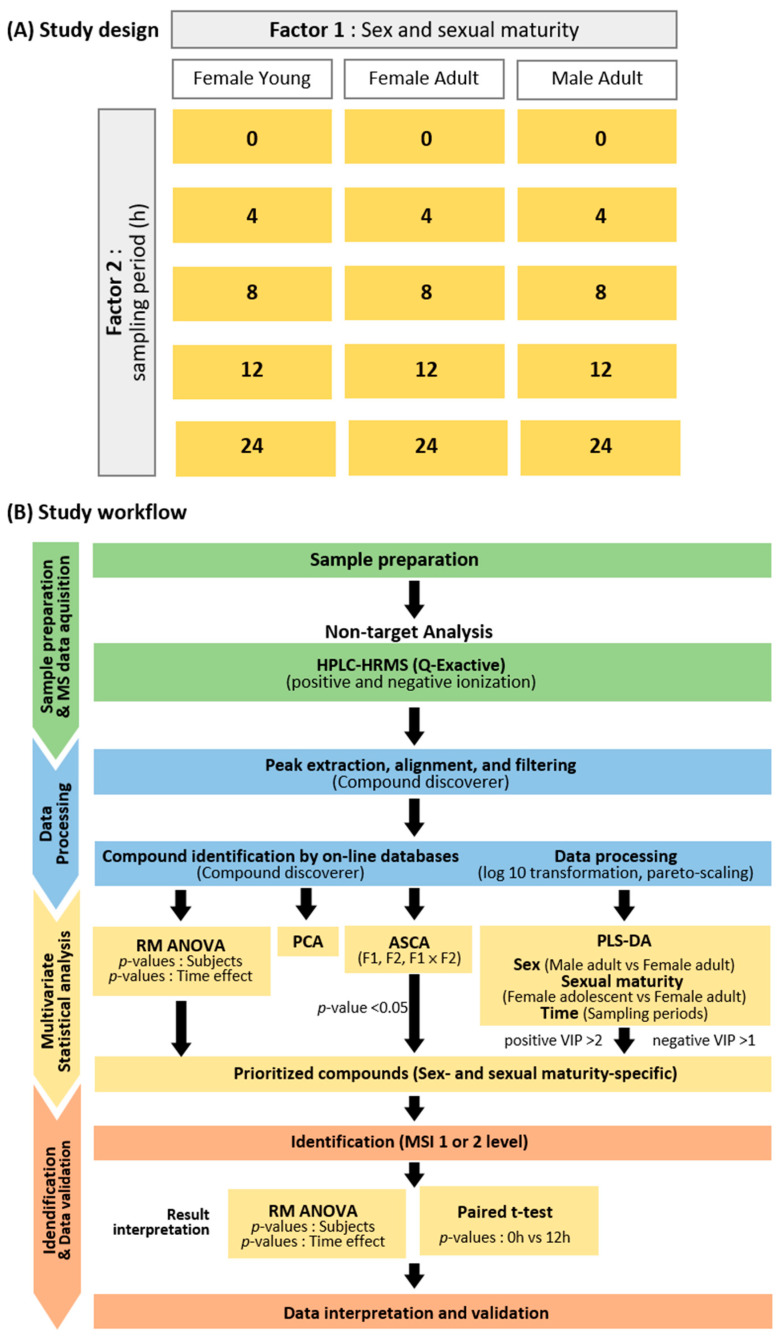
Overview of sample collection and study workflow. Brief information on the study design with sampling periods and groups (**A**); Workflow of the study from sample preparation through data acquisition and processing to identifying statistically significant compounds (**B**). Abbreviations: F1: Factor 1 (sex and sexual maturity), F2: Factor 2 (sampling period), HPLC: High-Performance Liquid Chromatography, HRMS: High-Resolution Mass Spectrometry: Triple Quadrupole Mass Spectrometer, PCA: principal component analysis, PLS–DA: partial least squares–discriminant analysis, VIP: Variable Importance in Projection.

Urine samples were collected based on two criteria: sex and sexual maturity (Factor 1) and the sampling period (h; Factor 2). Figure 1 provides detailed information regarding these criteria: Factor 1 (sex and sexual maturity) and Factor 2 (sampling period ((h)) are illustrated in part A, while part B outlines the study’s progression from sample preparation to metabolite identification and data validation. Various multivariate statistical analyses, including ASCA and PLS–DA, were employed to prioritize metabolites specific to sex and sexual maturity. These selected metabolites were then identified, and compounds graded as MSI 1 and 2 underwent further analysis using RM–ANOVA across all time points. A paired t-test was used to compare the control and the time point exhibiting the most contrasting metabolic profiles. This approach ensured the verification of biological toxic effects and validated significant metabolic perturbations according to sex- and sexual maturity-specific models.

### 2.2. Reagents and Materials

Bis(2-ethylhexyl) phthalate (DEHP), mono-2-ethylhexyl ester phthalate (MEHP), mono-(2-ethyl-5-hydroxyhexyl) phthalate (MEHHP), acetic acid, and olive oil were obtained from Sigma-Aldrich (St. Luis, MO, USA). Mono-(2-ethyl-5-oxohexyl) phthalate and deuterated versions of the above compounds were purchased from Toronto Research Chemicals (Toronto, ON, Canada). LC–MS grade methanol, acetonitrile, and water were purchased from JT Baker (Phillipsburg, NJ, USA).

### 2.3. Animals

To consider the sex-specific and sexual maturity-specific responses for single low-dose DEHP exposure, 5-week-old pre-mature adolescent female, 9-week-old matured female, and male rats were obtained from Orientbio (Gyeonggi, Republic of Korea). Within the stabilization period for a week, each rat was housed in a controlled environment under room temperature (23 ± 3 °C) with a humidity of 50 ± 20% and free access to water and food, with a 12 h light–dark cycle. Prior to administrating DEHP orally at 5 mg/kg of body weight, the rats were placed in metabolic cages and prohibited from eating overnight. Urine and plasma were collected five times (0, 4, 8, 12, and 24 h after DEHP administration). Ethical approval for all experimental procedures was obtained via the Institutional Animal Care and Use Committee of KIST (KIST-IACUC-2023-045).

### 2.4. Sample Preparation

The samples were prepared by mixing 20 μL of urine with 40 μL of a 1.5% acetic acid solution, including d4-MEHP, d4-MEOHP, and d4-MEHHP. Deuterated major DEHP metabolites were used as internal standards to monitor data quality and to ensure reproducibility. The resultant mixtures were vortexed for 5 min and centrifuged at 16,000× *g* for 10 min. The supernatants were analyzed via liquid chromatography and Q-Exactive and mass spectrometry. All urine samples were analyzed in a random order. Quality control (QC) samples were prepared by pooling the same volume of each sample and analyzing every set of ten analytical sample runs to validate the repeatability of the instrument.

### 2.5. Untargeted Metabolomics Experiment

Untargeted analysis of the rat urine after DEHP administration was performed using a vanquish liquid chromatographic system coupled with a Q-Exactive and mass spectrometer (Thermo Scientific, Carlsbad, CA, USA). Chromatographic separation was achieved on a Kinetex C18 100 Å LC column (100 mm × 2.1 mm, 2.6 µm, Phenomenex). The autosampler and column oven temperatures were 4 °C and 35 °C, respectively. Both mobile phases were prepared with 0.1% of formic acid in water as mobile phase A and 0.1% of formic acid in methanol as mobile phase B. The separation program, which flowed at 0.5 mL/min, was as follows: 0–1 min (2% B), 1–5 min (2–10% B), 5–10 min (10–50% B), 10–13 min (50–95% B), 13–15.5 min (95% B), 15.5–16 min (95–100% B), 16–18 min (100% B). The injection volume was 5 µL.

The tuning method was optimized using an H-ESI ion source for electrospray ionization with the following parameters. The positive and negative ion spray voltages were set at 4 kV and 3 kV, respectively. The sheath gas flow rate was 53 arbitrary units, the auxiliary gas flow rate was 14 arbitrary units, and the sweep gas flow rate was set to 3 arbitrary units. The S-Lens RF level was adjusted to 60, the capillary temperature was 269 °C, and the auxiliary gas heater was set to 438 °C. The resolution for the full mass scan was 70,000, the automatic gain control (AGC) was set at 3 × 10^6^, and the maximum injection time (IT) was 100 ms. The scanning mass range spanned from 70 to 1050 *m*/*z*. Subsequent data-dependent MS/MS operated at a resolution of 17,500, an AGC of 1 × 10^5^, a maximum IT of 50 ms, and a collision energy of 35 eV, with a 1 *m*/*z* mass isolation window.

### 2.6. Data Processing

The Compound Discoverer (version 3.0) was employed for peak integration, alignment, and correction, using the QC datasets and identifying metabolites via public MONA and HMDB spectral libraries. The feature table produced by the Compound Discoverer was then used for subsequent analyses. The features underwent filtering based on several criteria: ChemSpider (for both full and partial matches), prediction (full), MS/MS (with preferred ion), and a relative standard deviation (RSD) area % of QC less than 20% to ensure the capture of genuine molecular features. Before the statistical analysis, Pareto scaling and log transformations were applied to make the dataset a more Gaussian-type distribution [24].

### 2.7. Multivariate Data Analysis

Statistical evaluations of the results were performed using multivariate analyses, including principal component analysis (PCA), partial least squares–discriminant analysis (PLS–DA), analysis of variance–simultaneous component analysis (ASCA), and repeated-measures–ANOVA (RM–ANOVA). PCA and PLS–DA were conducted using SIMCA-P version 17 (Sartorius, Göttingen, Germany) to ensure dataset stability based on the QC samples and to visualize effects based on sex and sexual maturity when rats were exposed to DEHP. ASCA was conducted using MetaboAnalyst version 5.0. The metabolites with statistical significance, determined by PLS–DA (VIP > 2 for positive and VIP > 1 for negative) and ASCA (*p*-value < 0.05) in relation to sex and sexual maturity, were selected for metabolic pathway analysis. These selected metabolites underwent RM–ANOVA and were then used in the subsequent metabolic pathway and toxicity analyses. Moreover, a paired t-test was conducted to assess metabolic changes at a certain time point that exhibited the most distinct metabolic profiles compared with the baseline (prior to DEHP exposure), and the resulting *p*-value was calculated. In addition, the relative abundance of metabolites in DEHP and bile acid metabolisms was assessed using the RM–ANOVA. The RM–ANOVA was conducted using the ezANOVA package in R and SPSS (version 23). The metabolites that differentiated based on the paired t-test were determined using the pheatmap package in R.

### 2.8. Metabolic Pathway Analysis and Metabolites Identifications

Changes between groups were linked to specific metabolic pathways and analyzed via Ingenuity Pathway Analysis (IPA) and MetaboAnalyst, aiming to determine the relevant metabolomic pathways with a *p*-value threshold of <0.05. The list of metabolites utilized for toxic function and canonical pathway analysis in IPA was validated as level 1 or 2 of the metabolomics standard initiative (MSI) [25]. Twenty metabolites that matched not only accurate *m*/*z* but also the chromatographic retention time to the authentic standards were categorized as MSI 1. Additionally, metabolites with a 5 ppm mass tolerance were assigned as MSI 2. This validation compared the MS/MS spectrum with the Human Metabolome Database (http://www.hmdb.ca (accessed on 1 July 2022)) and MONA spectral library or authentic standards.

### 2.9. Liver Toxic Marker Test

The liver toxicity marker, plasma glutamate oxaloacetate transaminase (GOT1), was measured using a Luminex screen assay kit (RLI1MAG-92K) following the manufacturer’s instructions (Merck Millipore, Burlington, MA, USA). In brief, plasma was diluted at 1:25 with the assay buffer and incubated at 37 °C overnight. The standard stock was further diluted to create seven calibration points. Furthermore, 25 μL of each sample and calibrator were dispensed into pre-washed wells, and an equivalent volume of the background buffer was added. For purification, 25 μL of a well-mixed solution containing antibody-immobilized beads was added to each well. This mixture was then shaken and incubated for 2 h at room temperature. After this period, the plate was washed, and 25 μL of a detection antibody was added, followed by another 1 h of incubation at room temperature. An equal volume of Streptavidin–Phycoerythrin was added, and the solution was allowed to incubate for an additional 30 min. After incubation, each well was emptied and rinsed with a washing buffer. Finally, the beads were resuspended in 150 μL of the sheath fluid and analyzed using a Luminex analyzer (Luminex, Austin, TX, USA). The standard curve was generated using the best-fit model in MasterPlex QT 2010 software (MiraiBio, Hitachi, CA, USA).

## 3. Results

### 3.1. Untargeted Metabolomic Analysis of Rat Urine Samples

The initial feature table was refined based on several parameters, such as MSMS spectrum acquisition and stable detection, with an RSD of less than 20%, to ensure the credibility of these features. After filtering, 1734 ions were selected for negative ionization analysis, and 6390 ions were selected for positive ionization analysis. These selected ions were then subjected to statistical analysis. The PCA plots indicate that QC samples were consistently detected in both ionization modes throughout batch analysis (Figure S1). A clearer distinction was evident between sexes as opposed to levels of sexual maturity in female rats. The principal component (PC) indicated that differences in sex accounted for a more significant variation in metabolism than differences in age (Figure S1 (A): R2X[cum] 0.511 and Q2[cum] 0.478 for sex-specific model comparison in positive mode). However, the model for female sexual maturity revealed a greater overlap between the sexual maturity of groups. Nevertheless, distinct metabolic patterns emerged based on the hours after DEHP absorption (Figure S1 (B): R2X[cum] 0.415 and Q2[cum] 0.366 for sexual maturity in female groups in positive mode).

**Figure 2:**
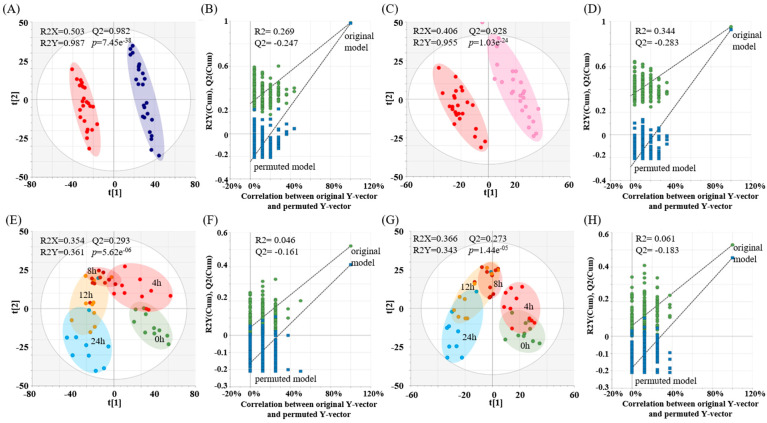
Partial least–squares discriminant analysis (PLS–DA) score plots and cross-validation plots of urinary metabolites analyzed in the positive ionization mode. Metabolic profiles in male adults and female adults (**A**) and female adolescents and female adults (**C**) are visualized with the blue circle denoting a male adult, pink circles denoting a female adolescent, and the red circle denoting a female adult. Time-dependent metabolic changes in male and female adult (**E**) and female adolescents and female adults (**G**) are depicted with multiple time points including 0 h (before DEHP exposure, green) and 4 h (bright red), 8 h (deep red), 12 h (orange), and 24 h (blue) after DEHP exposure. The PLS–DA models were evaluated using R2X, R2Y, Q2, and *p*-values and were cross-validated with a permutation test (*n* = 200). Cross-validation was qualified by intercepts of R2 and Q2 and showed high statistical significance wherein (**B**,**D**,**F**,**H**) matched with (**A**,**C**,**E**,**G**), respectively.

Given that this study was designed with multiple experimental factors (three groups investigating responses based on sex and sexual maturity and multiple time points for urinary excretion post-single-dose DEHP administration), PLS–DA, ASCA, and RM–ANOVA were employed to discern the major trends in metabolic changes relating to sex and maturity. Initially, PLS–DA, which offers VIPs, was used to identify variables that qualified. Differences in urine metabolite profiles across both sexes (mature males and females) and sexual maturity (female adults and adolescent females), as well as changes in metabolic patterns after DEHP exposure based on sex and maturity, were visually and statistically assessed via PLS–DA score plots (Figure 2A,C,E,G), each exhibiting statistically acceptable quality parameters. Each dataset revealed distinct metabolic patterns. Notably, metabolisms at 8 h and 12 h post-DEHP exposure contrasted starkly with the basal metabolism. The PLS–DA model’s statistical credibility was affirmed through cross-validation via permutation tests (*n* = 200) (Figure 2B,D,F,H). Additional PLS–DA datasets and cross-validation results can be found in Figure S2. The metabolites accounting for these observed differences were filtered using VIP values: >2 in the positive mode and >1 in the negative mode. The ion counts between sexes or between levels of sexual maturity were 580 and 496 in the positive mode, respectively, and 963 and 852 in the negative mode, respectively.

To ascertain the primary trends in metabolic changes and identify the underlying factor of time post-DEHP absorption, the ASCA, which is commonly utilized in metabolomics as a reference to the multivariate statistical method and RM–ANOVA was utilized. This approach facilitated a more profound analysis, highlighting sex- and sexual maturity-specific metabolic disturbances associated with DEHP absorption. The ASCA pinpointed important features that were characterized by high leverage and low SPE values. Specifically, significant features were identified between sexes or, when based on sexual maturity, amounted to 408 and 316 ions in the positive mode and 134 and 189 ions in the negative mode, respectively. Additionally, RM–ANOVA evaluated the metabolites perturbed specifically due to sex and sexual maturity, considering the time factor in their significance. From the RM–ANOVA results, the metabolites that exhibited significant differences based on sex and sexual maturity post-DEHP exposure accounted for 206 features for sex and 203 for sexual maturity in the positive mode and 162 and 151 in the negative mode, respectively. These features underwent further identification through authentic standards or public database matching and were scrutinized for their potential biological impacts, such as their toxicity or relevant pathways. Details regarding these metabolites, including retention time, ionization mode, RM–ANOVA results, and MSI level, are presented in Table 1 and Table 2 for sex and sexual maturity, respectively. DEHP’s urinary metabolites, including MEOHP, MEHHP, MECPP, and 5-oxo-MEHTP, exhibited significant variations both in time and across sex- and sexual maturity-specific models. The number of metabolites deemed significant within the groups, excluding their interaction with the time factor, totaled 23 metabolites for the sex-specific model (Table 1) and 10 metabolites for the sexual maturity-specific model (Table 2).

In the sex-specific model, 31 metabolites in the positive analytical mode and 42 metabolites in the negative analytical mode were found to change due to DEHP exposure. Conversely, the sexual maturity-specific model identified 40 metabolites in the positive mode and 46 metabolites in the negative mode, which were altered. Among these, several bile acids, including deoxycholic acid, glycocholic acid, muricholic acid, cholic acid, hyocholic acid, ketodeoxycholic acid, taurocholic acid, and hydroxycholic acid, along with organic acids, such as benzoic acid, citric acid, acetylneuraminic acid, salicylic acid, gentisic acid, 2-oxoadipic acid, caffeic acid, and 2-ethylhexanoic acid, were observed in both models. Additionally, steroid hormones, such as testosterone sulfate, hydroxypregnenolone sulfate, corticosterone, androstenedione, estriol, testosterone, progesterone, hydroxyprogesterone, cortolone, cortisone, cortisol, dihydroprednisolone, and dihydrocortisol, showed statistical differences in both models.

**Figure 3:**
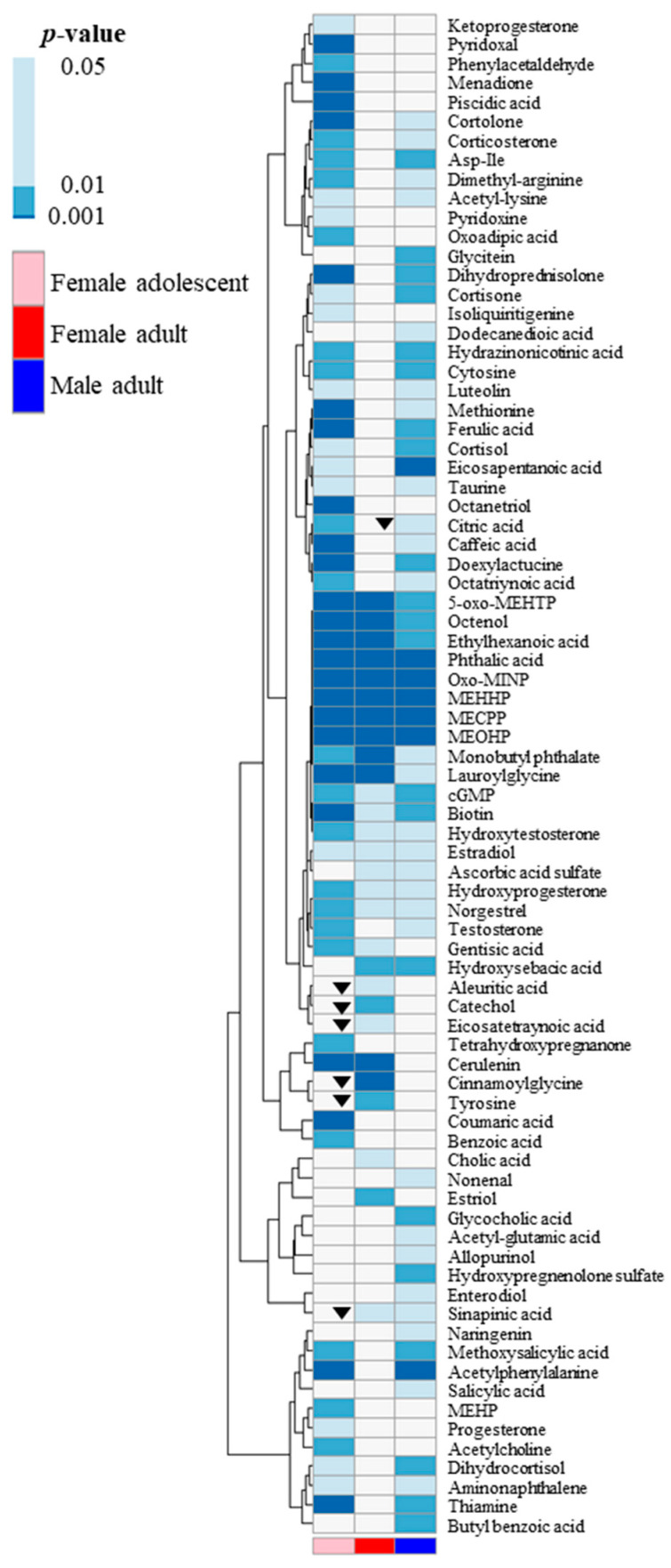
Heatmap of paired *t*-test *p*-values comparing metabolic profiles 12 h post-exposure to those before DEHP exposure, highlighting the most opposing metabolic profiles. *p*-values from the paired *t*-test are illustrated according to their range of significance. A triangle symbol indicates a decrease in metabolites at 12 h, while no symbol indicated an increase at 12 h when compared with the baseline. Female adolescents displayed the most pronounced metabolic shifts, followed by male adults, and then female adults.

The metabolites that were differentiated by RM–ANOVA underwent a paired t-test analysis to validate the significance of metabolic shifts between the baseline (prior to DEHP exposure) and 12 h post-DEHP exposure, which exhibited the most pronounced metabolic profile variations in PLS–DA (Figure 2E,G). Figure 3 presents a heatmap created based on the *p*-values for each group, with metabolites that decreased at the 12 h mark further highlighted with a triangle. The female adolescent group exhibited the most pronounced metabolic changes, followed by male and then female adults. Notably, most of the significantly altered steroid hormones, including ketoprogesterone, cortolone, corticosterone, dihydroprednisolone, hydroxytestosterone, hydroxyprogesterone, testosterone, and progesterone, displayed larger shifts in the female adolescent group. DEHP metabolites, such as MEHHP, MECPP, MEOHP, phthalic acid, and oxo-MINP, demonstrated noticeable increases across all groups.

### 3.2. Analysis of Key Metabolism Involved in DEHP Exposure

**Figure 4:**
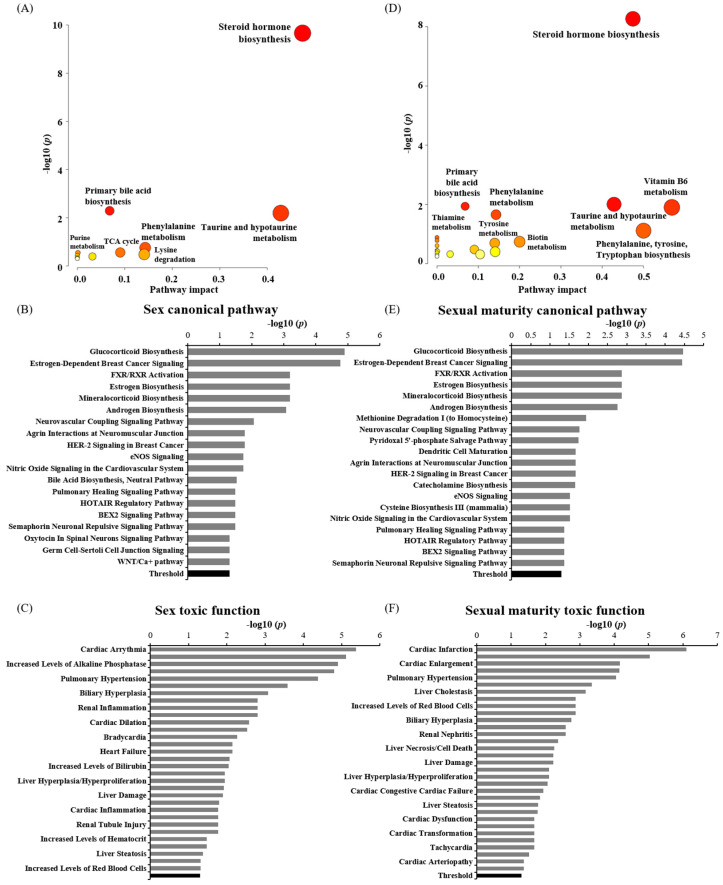
Statistically evaluated major pathways based on significantly altered metabolites between the male adult and female adult (**A**,**B**), and the female adolescent and female adult (**D**,**E**). The metabolites evaluated by RM–ANOVA were used to determine the most significantly impacted pathways, with *p*-values presented on the *Y*-axis and pathway impact values on the *X*-axis (**A**,**D**). The circle colors denote significance (*p*-value), while the size of each circle’s radius reflects the pathway’s impact values. The most significantly altered canonical pathways, as determined by IPA, are depicted in (**B**,**E**). Both group sets highlighted steroid hormones and bile acid biosynthesis as the primary metabolic pathways affected by DEHP exposure. Significant toxicological functions from IPA based on altered metabolites from RM–ANOVA are presented in (**C**,**F**). Heart, liver, and kidney damage emerged as the primary toxicological effects of DEHP administration.

The metabolites that showed significant changes across these three groups or at different time points post-DEHP exposure were analyzed for pathways using MetaboAnalyst and IPA software. These findings indicate that DEHP exposure predominantly impacted the steroid hormone biosynthesis, taurine and hypotaurine metabolism, phenylalanine metabolism, and primary bile acid biosynthesis pathways, as shown in Figure 4A,B,D,E. In the sexual maturity-specific model, the biosynthesis of phenylalanine, tyrosine, tryptophan, and vitamin B6 metabolism were closely linked to the metabolism activated by DEHP exposure. The IPA’s disease and functional analysis suggested that DEHP exposure primarily affected the metabolic processes associated with heart, liver, and kidney injuries, including cardiac infarction, pulmonary hypertension, liver cholestasis, and renal tubular injury (Figure 4C,F). For the sex-specific model, the most affected toxicological pathways due to DEHP were cardiac arrhythmia, increased alkaline phosphatase, pulmonary hypertension, and biliary hyperplasia. Meanwhile, for the sexual maturity-specific model, the most disrupted toxicity and functional features in IPA were cardiac infarction, cardiac enlargement, pulmonary hypertension, and liver cholestasis.

### 3.3. Analysis of Key Metabolic Pathway Involved in DEHP Exposure

**Figure 5:**
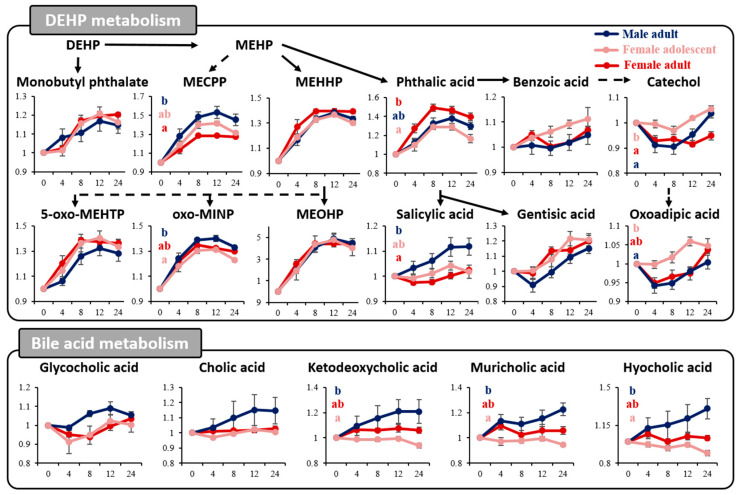
Metabolic pathways in rat urine influenced by the single administration of DEHP. Each metabolite was normalized via the basal condition of each rat (*n* = 5, per group), and the changes between these groups are displayed using line graphs. Black arrows and dotted arrows denote a direct and indirect relationship between these two metabolites. Statistical analysis was performed using RM–ANOVA, followed by the Scheffe post hoc analysis. Statistical significance is indicated only for those that showed no interaction among the groups and time factors. Different letters in the same column indicated significant differences at *p* < 0.05.

The fold change for all time points was analyzed based on the basal time point to account for inherent differences between the groups obtained before each rat’s DEHP exposure. This was then analyzed using RM–ANOVA, followed by Scheffe post hoc analysis (Figure 5). An examination of the metabolites associated with DEHP metabolism revealed that the levels of phthalic acid, MEOHP, MEHHP, MECPP, oxo-MINP, 5-oxo-MEHTP, and monobutyl phthalate exhibited consistent trends that increased and then decreased across all three groups. MEHP, a primary metabolite of DEHP, did not exhibit pronounced differences among the groups because it was rapidly generated from DEHP upon entering the body and then swiftly degraded for further catabolism [26]. However, the secondary metabolites of MEHP, such as MECPP and phthalic acid, displayed significantly varied expressions between female and male adult rats. Another indirect DEHP metabolite, salicylic acid, also exhibited a different pattern in their sex-specific comparison. DEHP metabolism end products, such as catechol and oxoadipic acid, were most abundant in the urine of female adolescent rats.

Beyond DEHP metabolism, primary bile acid biosynthesis and taurine and hypotaurine metabolism were also identified as significant pathways linked by sex and sexual maturity models. Notably, the bile acid family, including hyocholic acid, ketodeoxycholic acid, and muricholic acid, showed more substantial changes in male rats compared with the other groups, with clear differences evident when compared with female adolescent rats (Figure 5 bottom).

### 3.4. Liver Injury Biomarker GOT1 Disturbance Affected by DEHP

**Figure 6:**
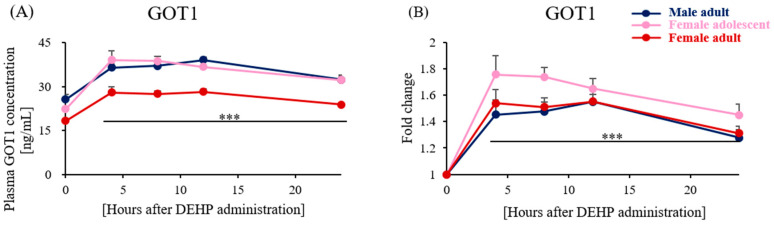
Time-dependent plasma concentrations of glutamate oxaloacetate transaminase 1 (GOT1), which are known as the liver damage biomarker, were analyzed 0, 4, 8, 12, and 24 h after single 5 mg/kg of DEHP administration in rats. The absolute concentration and fold change relative to basal levels, compared with the post-administration levels in each group, are both depicted in (**A**,**B**). When compared with the basal level, statistical significance was indicated at every time point with a *p*-value of less than 0.001 using ***, as assessed by a pairwise *t*-test with the Bonferroni adjustment.

To evaluate the liver injury stemming from metabolic changes caused by a single, low dose of DEHP, we analyzed the liver toxicity biomarker GOT1 in rats’ plasma. There were significant differences in the absolute GOT1 levels among the groups (Figure 6A; sex-specific model *p*-value: 8.22 × 10^−5^, sexual maturity-specific model *p*-value: 1.45 × 10^−3^); the fold changes did not exhibit significant variations (Figure 6B; sex-specific model *p*-value: 0.708, sexual maturity-specific model *p*-value: 0.138) in RM–ANOVA. Despite this, we observed a significant increase in GOT1 due to the administration of DEHP. Based on each rat’s basal time point, the fold change showed a clear increase when assessed by a pairwise t-test adjusted with the Bonferroni correction. However, no statistical difference among the three groups was detected. For instance, the one-way ANOVA *p*-value among the three groups was 0.068, 8 h post-DEHP exposure.

## 4. Discussion

Metabolomic analyses of urine samples from male adults, female adolescents, and female adult rats exposed to a low, single dose of di(2-ethylhexyl) phthalate (DEHP) were conducted using liquid chromatography–mass spectrometry (LC–MS). Although long-term DEHP exposures have been well documented, studies examining the metabolic response to a single, low dose of DEHP, particularly when comparing responses based on sex and sexual maturity, have been limited. Notably, even low dose concentrations cannot completely rule out the potential toxicity of DEHP. Previous studies have indicated that groups dosed with toxic substances can be exposed to risks relating to creatinine correction, as urinary creatinine levels can be influenced by factors such as disease, illness, age, and gender [27,28,29]. Specifically, in male adults, urine creatinine significantly increased 24 h after DEHP administration when compared with the basal, 4 h, and 8 h measurements. Thus, correcting using this value might lead to inaccuracies when interpreting the actual metabolic dynamics (Figure S3). Hence, untargeted raw data were adjusted based on the total peak area in each sample using log and Pareto scaling without any creatinine correction [27,30]. This adjusted dataset was further assessed for validation and extended identification to determine the effects of DEHP exposure across different sexes and stages of sexual maturity in rats more accurately.

Exposure to DEHP during the developmental stages could intensify its detrimental health effects due to the immature metabolic processes present in children. Several studies have linked DEHP exposure to variations in the onset of puberty, though findings vary depending on gender. For instance, female rats exposed to DEHP via inhalation experienced a notably earlier onset of puberty [31]. Conversely, male rats given dosages exceeding 9 mg/kg of body weight daily for 28 days displayed a delay in the onset of puberty. Notably, female rats exposed to equivalent DEHP levels showed no discernible differences from the standard rat population [11]. These observations imply that DEHP exposure might have a more pronounced impact on the reproductive function of male rats. However, the exact molecular mechanisms driving these effects remain elusive. As such, our study aimed to explore the contrasting influences of DEHP on both male and female rats after single, low-dose DEHP exposure. We also attempted to determine the molecular pathways responsible for these developmental discrepancies, considering both sex and stages of sexual maturity.

Regarding the specific effects of DEHP based on sex and sexual maturity, our results highlight pronounced differences in metabolic urine profiles among adult male and female rats and between the adolescent and adult female rats after single DEHP ingestion. The PCA and PLS–DA indicated a peak alteration at 12 h relative to the basal points, which was attributed to the prominent disruption caused by the urinary excretion of DEHP metabolites (Figure 2). Moreover, RM–ANOVA revealed statistical variations in DEHP metabolites across sex and sexual maturity models (Table 1 and Table 2). The metabolites that displayed statistical significance in both the sex and sexual maturity models were closely associated with pathways such as steroid hormone biosynthesis, primary bile acid biosynthesis, and taurine and hypotaurine metabolism (Figure 4). In female adolescent rats and male adult rats, several steroid hormones, including corticosterone, cortolone, hydroxytestosterone, hydroxyprogesterone, and testosterone, exhibited a notable increase 12 h post-DEHP exposure. These hormonal changes were assessed using the paired t-test and are illustrated in Figure 3. Our findings are consistent with prior studies suggesting that prepubertal DEHP exposure leads to testicular injury when steroid hormone biosynthesis-related transcriptome is disrupted [32]. Beyond steroid hormones, these data indicate that perturbations in intermediate metabolites in male rats might trigger the upregulation of bile acid biosynthesis. Contemporary studies have demonstrated that DEHP disrupts the cholesterol balance by influencing bile acid metabolism through gut microbiota composition changes [18]. For instance, in our study, significant increases in hyocholic acid and ketodeoxycholic acid were observed in male rats (Figure 5). This aligns with past research suggesting that excreted hyocholic acid can mitigate liver injury via modulating lipid metabolism [33] and that ketodeoxycholic acid levels rise in cases of liver damage [34,35]. However, when analyzing data based on sexual maturity-specific comparisons, no significant fold change differences in major metabolites were evident. There was a lack of observable variations in response to a single DEHP administration at a dosage of 5 mg/kg of body weight in sexually mature subjects; specific responses only emerged under consistent and substantial DEHP exposure. In this investigation, discerning subtle differences due to sexual maturity solely from a one-time DEHP dose of 5 mg/kg body weight proved inadequate when highlighting the distinct reactions between the pre-and post-puberty stages.

Our data demonstrated that DEHP exposure might elevate the risk of heart, liver, and kidney damage (Figure 4C,F). This suggests that DEHP has the potential to disrupt liver homeostasis. To further understand this, we evaluated plasma GOT1 levels at five distinct time points: the basal point and 4, 8, 12, and 24 h post-DEHP administration. Although the absolute quantity did not distinctly delineate vulnerability across these groups, there was a significant increase in the hepatotoxicity marker and GOT1 post-DEHP exposure in all the rats (Figure 6A). Moreover, when compared with the basal point, the fold change in GOT1 at 8 h post-DEHP administration exhibited the most pronounced difference among these groups. However, no significant variation was detected using the one-way ANOVA test (*p*-value 0.068). The current study draws a potential link between the elevation in the plasma GOT1 concentration and alterations in bile acid metabolism following a single administration of 5 mg/kg of body weight for DEHP. However, a deeper exploration is necessary to understand this relationship comprehensively. Integrating insights from previous toxicological studies [2,36] and our findings, we posit that heightened GOT1 levels and disrupted bile acid metabolism, even after single, low-dose DEHP administration, might predominantly stem from DEHP’s hepatotoxicity. However, our study is not without its limitations. Because we did not consistently monitor a blank control group across all the models and primarily focused on the sex- and sexual maturity-specific responses to DEHP, assuming the basal point of each rat served as a control for the treatment and additional research was crucial to ascertain the extent of hepatotoxicity induced by DEHP. Additionally, while the untargeted analysis identified steroid hormone and bile acid biosynthesis as pathways primarily affected by DEHP exposure, absolute quantification could provide a more detailed understanding. Nevertheless, our research lays the groundwork for future investigations, especially those examining DEHP-induced hepatotoxicity and disruptions in bile acid metabolism over multiple time measurements.

## 5. Conclusions

In this study, the sex- and sexual maturity-specific effects of low-dose DEHP were assessed using integrated LC–MS-based untargeted metabolomics analysis. These findings indicate that the sex-specific model presented a more pronounced distinction in response to a single low dose of DEHP when compared with the sexual maturity-specific model. DEHP appears to exert its effects in a manner that is dependent on sex and sexual maturity, particularly impacting steroid hormone and bile acid biosynthesis pathways. This suggests that DEHP exposure might interfere with lipid and liver metabolisms. Additionally, these experiments indicate a potential link between DEHP’s disruption of steroid hormone biosynthesis and bile acid pathways and its induction of hepatotoxicity, as evidenced by increased plasma GOT1 levels. Although further research is necessary to elucidate the specific mechanisms driving DEHP’s differential impacts based on sex and sexual maturity, this study offers a deeper insight into the metabolic alterations triggered by single, low-concentration DEHP exposure.

## Figures and Tables

**Table 1:** List of urinary metabolites showing significant variations based on sex and post-DEHP exposure time.

Mode	Name	Experimental Mass (*m*/*z*)	RT (Min)	*p*-Value (Sex)	*p*-Value (Time)	*p*-Value (s x t)	MSI	Mode	Name	Experimental Mass (*m*/*z*)	RT (Min)	*p*-Value (Sex)	*p*-Value (Time)	*p*-Value (s x t)	MSI
(ESI)-	Benzoic acid	121.0282	9.27	>0.05	5.62E−03	>0.05	1	(ESI)+	Allopurinol	137.0460	2.21	2.04E−03	1.20E−04	3.44E−02	1
	Taurine	124.0061	0.54	4.33E−05	2.14E−02	>0.05	1		Acetylcholine	146.1176	0.44	1.33E−06	1.16E−05	>0.05	1
	Citric acid	191.0187	0.73	3.71E−03	3.87E−04	>0.05	1		Menadione	173.0600	12.48	>0.05	8.91E−07	>0.05	1
	Monobutyl phthalate	221.0814	11.99	3.92E−03	1.16E−07	4.07E−02	1		Caffeic acid	181.0498	7.59	>0.05	1.74E−08	>0.05	1
	Naringenin	271.0612	11.19	>0.05	5.15E−07	>0.05	1		Luteolin	287.0552	11.05	>0.05	5.15E−07	>0.05	1
	MEHP	277.1443	13.29	>0.05	>0.05	>0.05	1		Corticosterone	347.2216	12.84	3.85E−09	1.94E−02	>0.05	1
	MEOHP	291.1237	11.93	8.73E−04	1.94E−06	>0.05	1		Cholic acid	391.2842	12.98	>0.05	1.40E−03	>0.05	1
	MEHHP	293.1393	12.15	1.54E−03	5.58E−06	>0.05	1		Hyocholic acid	817.5818	12.85	5.71E−04	>0.05	>0.05	1
	Acetylneuraminic acid	308.0984	0.43	1.64E−05	5.42E−03	>0.05	1		Phenylacetaldehyde	121.0652	9.69	2.75E−04	1.48E−02	>0.05	2
	cGMP	344.0400	1.44	>0.05	4.09E−09	>0.05	1		Octenol	129.1277	11.55	1.85E−07	1.50E−04	2.04E−02	2
	Deoxycholic acid	391.2854	13.60	>0.05	1.65E−02	>0.05	1		Salicylic acid	139.0392	8.38	2.05E−05	1.68E−04	8.44E−03	2
	Glycocholic acid	464.3013	12.11	3.10E−09	4.82E−04	4.55E−06	1		2-nonenal	141.1276	10.33	1.66E−04	1.49E−02	1.11E−02	2
	Catechol	109.0281	2.39	>0.05	4.33E−05	2.15E−02	2		2-Aminonaphthalene	144.0810	6.09	>0.05	3.21E−06	>0.05	2
	Gentisic acid	153.0216	2.77	>0.05	3.92E−06	>0.05	2		2-Ethylhexanoic acid	145.1225	8.68	>0.05	1.63E−08	>0.05	2
	2-oxoadipic acid	159.0288	0.77	>0.05	2.32E−04	>0.05	2		Phthalic acid	167.0341	12.22	5.76E−05	3.21E−06	5.00E−02	2
	Methoxysalicylic acid	167.0339	8.58	>0.05	1.92E−07	>0.05	2		Butyl benzoate	179.1069	8.24	9.01E−06	1.32E−03	1.37E−02	2
	Acetyl-glutamic acid	188.0554	0.50	1.44E−02	>0.05	9.51E−03	2		Acetyl lysine	189.1234	0.47	1.48E−04	1.69E−04	2.52E−02	2
	Acetylphenylalanine	206.0816	8.55	6.03E−03	4.57E−09	5.64E−03	2		Xanthoxyline	197.0784	5.29	4.81E−04	>0.05	1.51E−02	2
	Sinapinic acid	223.0606	8.50	3.58E−06	1.72E−02	1.80E−04	2		Dimethyl-arginine	203.1504	0.46	3.54E−06	1.83E−05	2.14E−03	2
	Isoliquiritigenin	255.0661	10.68	2.98E−02	7.85E−04	2.25E−02	2		Cerulenin	224.1283	11.70	4.92E−02	3.43E−07	4.10E−07	2
	Estradiol	271.1703	11.63	4.59E−08	3.62E−08	3.15E−04	2		Lauroylglycine	258.2066	12.61	3.57E−07	1.50E−04	8.79E−04	2
	Glycitein	283.0611	11.29	>0.05	7.19E−07	3.80E−02	2		Adenosine	268.1040	1.71	2.94E−03	2.70E−06	1.40E−02	2
	5-oxo-MEHTP	291.1238	12.04	2.19E−04	1.79E−07	>0.05	2		Naringenin	273.0759	11.45	>0.05	1.71E−02	>0.05	2
	oxo-MINP	305.1602	12.15	2.01E−04	8.84E−06	>0.05	2		Androstenedione	287.2006	12.14	1.44E−09	7.79E−05	>0.05	2
	MECPP	307.1186	11.36	1.66E−03	1.93E−06	>0.05	2		Estriol	289.1802	12.32	2.19E−04	5.65E−04	>0.05	2
	Enterodiol	301.1443	11.23	1.04E−05	4.44E−02	1.69E−02	2		Testosterone	289.2162	11.99	1.87E−06	3.41E−08	>0.05	2
	Eicosapentanoic acid	301.2172	12.32	1.33E−09	6.52E−04	1.39E−02	2		Eicosatetraynoic acid	297.1851	12.75	1.29E−08	4.07E−04	7.14E−07	2
	Testosterone sulfate	367.1581	12.91	4.26E−06	>0.05	>0.05	2		Hydroxytestosterone	305.2112	11.27	6.64E−07	>0.05	6.41E−03	2
	Ketodeoxycholic acid	405.2644	12.92	1.33E−02	5.77E−03	>0.05	2		Norgestrel	313.2163	11.78	1.47E−05	3.59E−05	1.18E−02	2
	Muricholic acid	407.2800	12.94	>0.05	3.56E−03	>0.05	2		Progesterone	315.2319	12.91	2.98E−09	>0.05	>0.05	2
	17-Hydroxypregnenolone sulfate	411.1845	13.09	4.42E−08	>0.05	>0.05	2		Pregnenolone	317.2475	12.45	5.24E−05	>0.05	>0.05	2
	Hydroxycholic acid	423.2752	12.22	>0.05	9.38E−03	>0.05	2		Ketoprogesterone	329.2112	11.13	3.92E−09	6.15E−06	8.61E−04	2
									17-Hydroxyprogesterone	331.2268	12.25	1.35E−05	2.09E−02	4.76E−02	2
									Cortolone	349.2373	12.68	1.14E−05	1.80E−03	8.55E−03	2
									Cortisone	361.2010	11.84	3.06E-10	2.34E−05	2.59E−02	2
									Cortisol	363.2166	11.97	6.43E−07	3.32E−07	4.47E−02	2
									Dihydroprednisolone	363.2166	11.83	3.37E−07	4.47E−04	4.04E−02	2
									Dihydrocortisol	365.2324	10.48	1.65E−07	5.36E−03	7.63E−03	2
									Ketodeoxycholic acid	407.2792	12.80	7.46E−06	1.54E−02	>0.05	2
									17-Hydroxypregnenolone sulfate	413.1992	13.46	1.21E−07	>0.05	>0.05	2
									Glycocholic acid	466.3164	12.09	1.70E−08	3.65E−03	1.78E−02	2
									Taurocholic acid	516.2992	13.84	4.31E−03	6.52E−03	>0.05	2

**Table 2:** List of urinary metabolites exhibiting significant variations based on sexual maturity and post-DEHP exposure time.

Mode	Name	Experimental Mass (*m*/*z*)	RT (Min)	*p*-Value (Sexual Maturity)	*p*-Value (Time)	*p*-Value (a x t)	MSI	Mode	Name	Experimental Mass (*m*/*z*)	RT (Min)	*p*-Value (Sexual Maturity)	*p*-Value (Time)	*p*-Value (a x t)	MSI
(ESI)-	Benzoic acid	121.0282	9.27	3.59E−02	1.43E−04	>0.05	1	(ESI)+	Allopurinol	137.0460	2.21	>0.05	>0.05	4.94E−02	1
	Taurine	124.0061	0.54	1.89E−02	1.76E−02	>0.05	1		Acetylcholine	146.1176	0.44	>0.05	3.98E−06	>0.05	1
	Citric acid	191.0187	0.73	>0.05	9.95E−03	3.48E−02	1		Menadione	173.0600	12.48	2.18E−03	8.65E-10	5.56E−03	1
	Monobutyl phthalate	221.0814	11.99	>0.05	6.90E-14	>0.05	1		Caffeic acid	181.0498	7.59	7.31E−03	4.15E-11	4.20E−02	1
	Naringenin	271.0612	11.19	>0.05	8.80E−04	>0.05	1		Luteolin	287.0552	11.05	4.06E−03	1.08E−07	>0.05	1
	MEHP	277.1443	13.29	>0.05	>0.05	4.19E−02	1		Corticosterone	347.2216	12.84	2.06E−03	4.13E−07	2.33E−02	1
	MEOHP	291.1237	11.93	>0.05	1.88E−07	>0.05	1		Cholic acid	391.2842	12.98	>0.05	5.57E−03	1.66E−02	1
	MEHHP	293.1393	12.15	>0.05	4.60E−06	>0.05	1		Hyocholic acid	817.5818	12.85	>0.05	8.51E−03	8.05E−03	1
	Acetylneuraminic acid	308.0984	0.43	>0.05	1.64E−05	3.27E−03	1		Cytosine	112.0510	4.31	9.30E−05	7.43E−06	1.25E−02	2
	cGMP	344.0400	1.44	>0.05	1.12E−07	>0.05	1		Phenylacetaldehyde	121.0652	9.69	>0.05	1.17E−04	2.92E−03	2
	Deoxycholic acid	391.2854	13.60	>0.05	4.77E−02	>0.05	1		Octenol	129.1277	11.55	>0.05	2.36E−05	>0.05	2
	Glycocholic acid	464.3013	12.11	5.06E−04	>0.05	>0.05	1		Octatriynoic acid	133.0287	2.33	1.57E−02	1.45E-10	3.48E−02	2
	Catechol	109.0281	2.39	3.27E−03	6.70E−03	5.47E−03	2		Salicylic acid	139.0392	4.32	>0.05	1.33E−03	>0.05	2
	trans-Cinnamic acid	147.0440	8.30	1.12E−02	>0.05	3.72E−02	2		2-nonenal	141.1276	9.97	2.75E−04	1.01E−02	3.49E−02	2
	Gentisic acid	153.0216	2.77	>0.05	4.41E−07	>0.05	2		2-Aminonaphthalene	144.0810	8.61	>0.05	4.65E−03	7.25E−04	2
	2-oxoadipate	159.0288	0.77	1.67E−02	3.05E−04	2.82E−02	2		2-Ethylhexanoic acid	145.1225	8.68	>0.05	2.22E-12	>0.05	2
	Methyladipic acid	159.0652	8.39	1.54E−02	5.59E−03	3.40E−02	2		Methionine	150.0582	0.57	>0.05	4.94E−07	4.53E−02	2
	Coumaric acid	163.0390	9.45	8.51E−03	1.02E-10	1.94E−02	2		Hydrazinonicotinic acid	154.0612	4.31	5.90E−05	8.75E−06	1.40E−02	2
	Pyridoxine	168.0656	0.72	2.56E−02	2.40E−03	3.75E−02	2		Octanetriol	163.1330	9.18	1.21E−02	5.88E-10	1.85E−02	2
	Sebacic acid	201.1124	11.54	8.34E−03	3.33E−02	2.26E−03	2		Phthalic acid	167.0341	11.06	2.40E−02	1.24E-13	>0.05	2
	Cinnamoylglycine	204.0659	9.48	>0.05	1.18E−06	9.06E−03	2		Pyridoxal	168.0657	0.59	>0.05	3.49E−08	1.70E−04	2
	Acetylphenylalanine	206.0816	8.55	8.41E−03	1.16E−03	4.96E−02	2		trans-Ferulic acid	177.0549	9.25	>0.05	5.62E−05	4.50E−02	2
	Hydroxysebacic acid	217.1075	10.63	>0.05	3.64E−03	6.62E−03	2		Tyrosine	182.0814	7.58	1.85E−03	1.35E−07	7.40E−05	2
	Dodecanedioic acid	229.1440	11.20	2.22E−04	2.71E−03	2.36E−03	2		Acetyl lysine	189.1234	0.47	>0.05	8.46E−04	3.40E−02	2
	Asp-ile	245.1139	0.91	6.79E−03	8.67E−09	4.13E−02	2		Xanthoxyline	197.0784	5.29	>0.05	3.07E−03	6.51E−03	2
	Ascorbic acid sulfate	254.9814	1.03	>0.05	>0.05	4.94E−03	2		Dimethyl-arginine	203.1504	0.46	>0.05	2.95E−07	5.00E−05	2
	Piscidic acid	255.0507	6.73	2.60E−02	5.47E−09	1.05E−02	2		Oxododecanoic acid	215.1645	10.84	1.30E−04	1.56E−02	4.04E−04	2
	Isoliquiritigenin	255.0661	10.68	3.22E−02	1.16E−02	2.07E−02	2		Biotin	245.0955	8.07	2.10E−03	7.34E-12	4.55E−02	2
	Deoxylactucin	259.0975	9.97	3.43E−02	7.95E−06	4.01E−02	2		Thiamine	265.1117	0.46	4.33E−02	1.48E−08	1.74E−02	2
	Estradiol	271.1703	11.63	>0.05	8.28E−07	4.43E−02	2		Adenosine	268.1040	1.71	>0.05	1.22E−04	1.46E−02	2
	5-oxo-MEHTP	291.1238	12.04	4.95E−02	1.66E−07	>0.05	2		Naringenin	273.0759	11.45	>0.05	>0.05	>0.05	2
	oxo-MINP	305.1602	12.15	>0.05	3.84E−05	>0.05	2		Androstenedione	287.2006	12.14	1.97E−05	>0.05	1.73E−02	2
	Aleuritic acid	303.2176	11.44	1.23E−02	7.88E−05	9.30E−03	2		Estriol	289.1802	12.32	2.13E−03	3.77E−03	>0.05	2
	MECPP	307.1186	11.36	7.45E−03	3.89E−06	>0.05	2		Testosterone	289.2162	11.99	>0.05	3.26E−06	1.56E−04	2
	Testosterone sulfate	367.1581	12.91	>0.05	>0.05	>0.05	2		6-hydroxytestosterone	305.2112	11.99	>0.05	2.78E−08	9.90E−04	2
	Tetrahydroxypregnanone	365.2330	11.93	3.18E−02	4.75E−05	7.24E−07	2		Norgestrel	313.2162	11.99	>0.05	1.09E−07	4.17E−04	2
	Acetyl adenylate	388.0649	0.45	>0.05	2.72E−06	1.18E−04	2		Progesterone	315.2319	12.91	7.79E−05	>0.05	>0.05	2
	Ketodeoxycholic acid	405.2644	12.92	>0.05	>0.05	1.70E−02	2		Pregnenolone	317.2475	12.45	>0.05	>0.05	>0.05	2
	Muricholic acid	407.2804	12.29	>0.05	>0.05	3.75E−02	2		Ketoprogestrone	329.2112	11.13	1.01E−04	3.35E−03	1.85E−03	2
	17-Hydroxypregnenolone sulfate	411.1845	13.09	>0.05	3.02E−02	4.36E−03	2		17-Hydroxyprogesterone	331.2268	11.99	>0.05	1.07E−07	6.14E−04	2
	Hydroxycholic acid	423.2752	12.22	3.04E−02	>0.05	3.30E−02	2		Cortolone	349.2373	12.42	1.23E−05	2.10E−06	5.48E−03	2
									Cortisone	361.2010	11.84	>0.05	2.22E−03	3.19E−02	2
									Cortisol	363.2165	9.20	>0.05	4.68E−08	5.30E−03	2
									Dihydroprednisolone	363.2166	11.83	9.57E−04	2.30E−05	8.03E−03	2
									Dihydrocortisol	365.2324	10.48	>0.05	1.10E−04	5.72E−03	2
									Ketodeoxycholic acid	407.2793	12.16	>0.05	>0.05	1.12E−02	2
									Glycocholic acid	466.3164	12.09	6.38E−03	6.81E−03	>0.05	2
									Taurocholic acid	516.2993	12.79	>0.05	1.17E−03	>0.05	2

## Data Availability

Data are available upon request to the corresponding author.

## Supplementary Materials

The following supporting information can be downloaded at: https://www.mdpi.com/article/10.3390/toxics11090794/s1, Figure S1: PCA score plot considering time-dependent manner after DEHP exposure; Figure S2: PLS–DA score plot and those cross-validation plots of urinary metabolites analyzed in negative ionization mode; Figure S3: Relative abundance of urinary creatinine obtained from non-target analysis (A) and its fold change based on basal point (B).
