# Supplementary material for: Untargeted Metabolomics Analysis Reveals Toxicity Based on the Sex and Sexual Maturity of Single Low-Dose DEHP Exposure
Source: Toxics. 2023 Sep 20;11(9):794. doi: 10.3390/toxics11090794 (PMC10538058; doi:10.3390/toxics11090794)
Supplement: Supplementary file 1 [file toxics-11-00794-s001.zip › toxics-2583710-supplementary.pdf]

toxics

MDPI

Supplementary Materials: Untargeted Metabolomics Analysis Reveals Toxicity by Sex and Sexual Maturity of the Single Low-Dose DEHP Exposure

Hyeon-Jeong Lee, Jonghwa Jin, Yoondam Seo, Inseon Kang, Junghyun Son, Eugene C Yi, Hophil Min

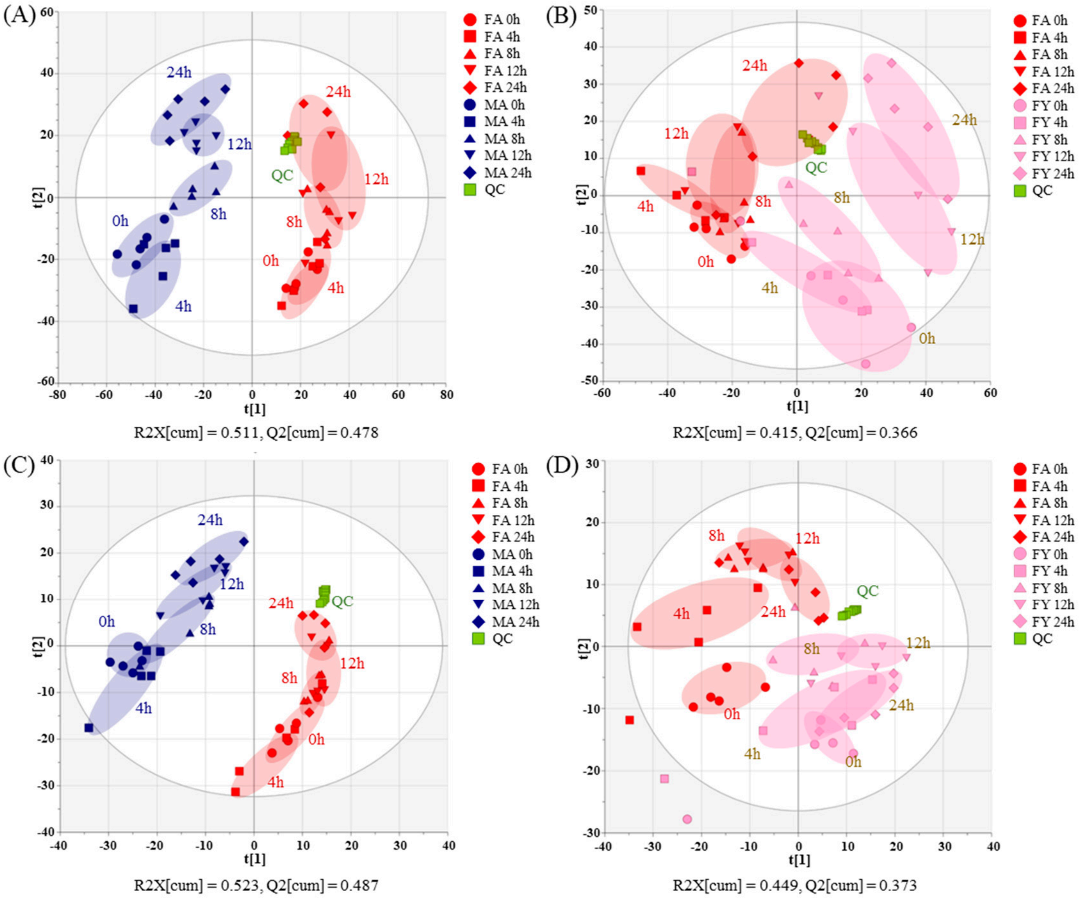

Figure S1. PCA score plot considering time dependent manner after DEHP exposure.
(A) and (B) are data acquired by positive mode and (C) and (D) are analyzed using negative mode. All quality control (QC) samples are observed to be clustered correctly.

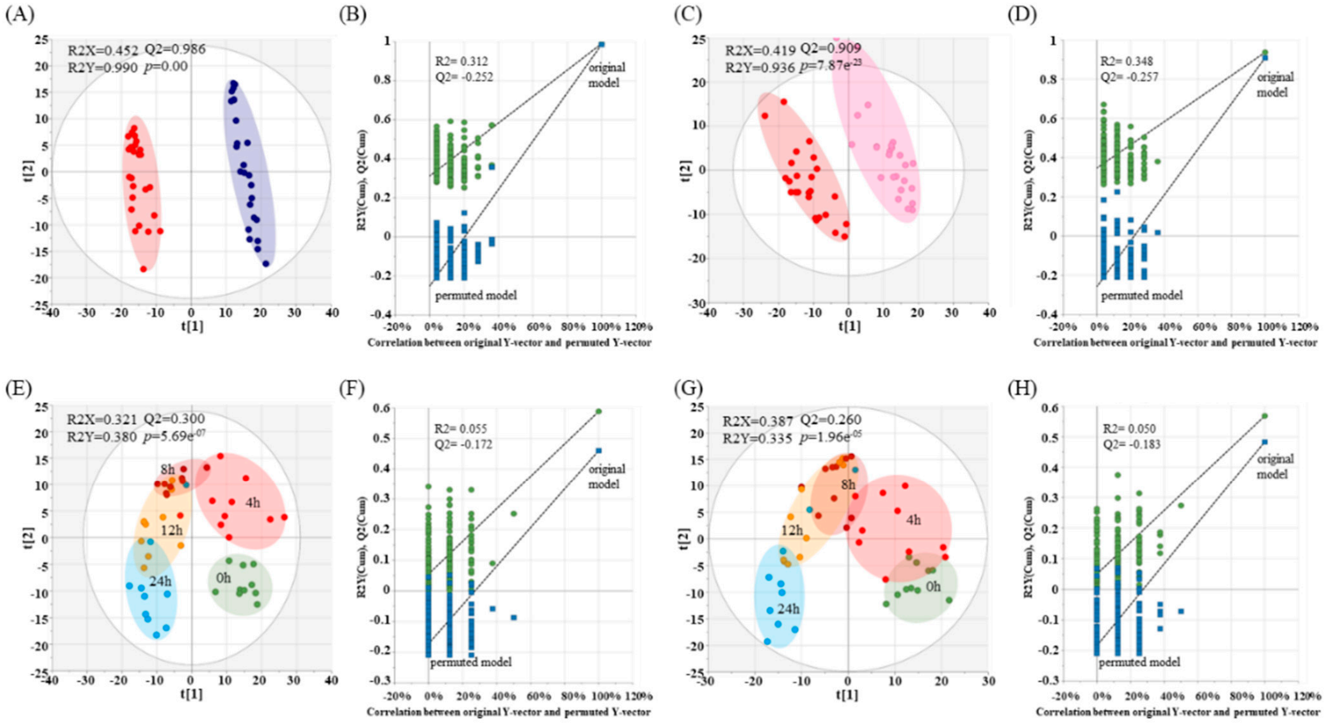

Figure S2. PLS-DA score plot and corresponding cross-validation plots of urinary metabolites, analyzed under negative ionization mode.

For male adults versus female adults: score plot (A) and its time-dependent metabolites post-DEHP absorption (E). For female adolescent versus female adults: score plot (C) and its time-dependent metabolites post-DEHP administration (G). The PLS-DA models were assessed using R2X, R2Y, Q2, and *p*-values and were cross-validated with a permutation test (n = 200). Cross-validation results, depicted by intercepts of R2 and Q2, indicate high statistical significance. Plots (B), (D), (F), and (H) correspond to the validation of (A), (C), (E), and (G), respectively.

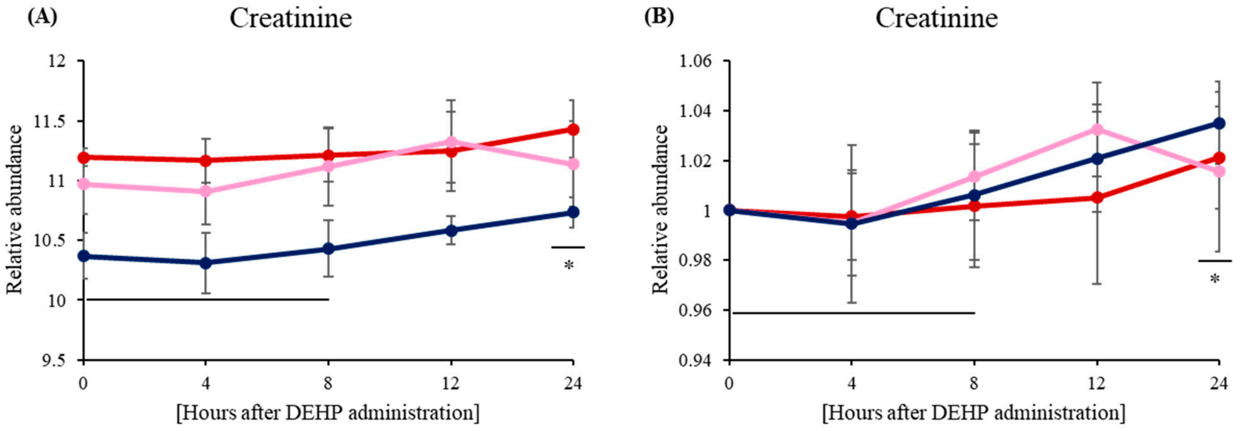

Figure S3. Relative abundance of urinary creatinine obtained from non-target analysis (A) and its fold change based on basal point (B).

RM-ANOVA determined significant difference of urinary creatinine between female adult and male adults (*p*-value 1.55e^{-5}). According to pairwise comparisons using paired t-test with bonferroni adjustment, relative abundance and fold changes showed significant differences between 24 h and other time points (0, 4, and 8 h) in sexual model.
